# A Moderated Mediation Model of Emotional Engagement in the Development of Emotional Exhaustion: The Moderating Role of Emotional Resources

**DOI:** 10.3389/fpsyg.2022.878415

**Published:** 2022-04-28

**Authors:** Ling Hu, Tai-Wei Chang, Yue-Shi Lee, Chien-Hsiang Huang

**Affiliations:** ^1^Department of Finance, Hsing Wu University, New Taipei City, Taiwan; ^2^Graduate School of Resources Management and Decision Science, National Defense University, Taipei City, Taiwan; ^3^Department of Computer Science and Information Engineering, Ming Chuan University, Taoyuan City, Taiwan; ^4^General Education Center, Chihlee University of Technology, New Taipei City, Taiwan

**Keywords:** leadership, emotional engagement, emotional exhaustion, emotional regulation, emotional resources

## Introduction

Contemporary companies should inspire their employees to invest their resources in the job (e.g., emotional engagement) through organizational management mechanisms (e.g., leadership) and also should pay attention to mitigate their negative behaviors (e.g., emotional exhaustion) at the same time (Zeng et al., 2020; Stoyanova and Stoyanov, 2021). Indeed, previous studies have paid attention to exploring the antecedent of emotional exhaustion, but, to date, there is no study to examine how leadership can increase emotional engagement, which in turn, reduce emotional exhaustion with the moderating effect of emotional resources. In common practice, emotional exhaustion is not a trivial concept, because it affects many economic factors, such as negative work behavior (Ding et al., 2018) and turnover intention (Lee et al., 2019). Past research of emotional exhaustion almost uses the job demand-resources model to examine intervention strategies, such as social support (Baeriswyl et al., 2016), job resources (Moreno-Jiménez et al., 2021), and emotional labor (Zhu et al., 2021), but little study employs emotional regulation perspective to explore the intervention strategies of emotional exhaustion. Indeed, emotional exhaustion means a feeling of exhaustion for emotional resources (Maslach et al., 2001), so emotional engagement should be a key antecedent to emotional exhaustion. Therefore, this research borrows emotional engagement from Kahn's theory (Kahn, 1990) to be a key antecedent variable of emotional exhaustion. Kahn's theory (Kahn, 1990) believes that an individual invests cognitive resources (cognitive engagement), emotional resources (emotional engagement), and physical resources (physical engagement) into role performance because the individual obtains meaningfulness, safety, and confidence from his or her job. In addition, a leader can shape a subordinate's value to meet a company value, and the company value can be delivered in the leadership process, which will increase the subordinate's meaningfulness of job. Therefore, leadership will increase emotional engagement, and then will decrease emotional exhaustion.

Although past research has paid attention to emotional resources in the multidisciplinary field (e.g., Wright et al., 2013; Liu et al., 2019; Ramchandran et al., 2020), the boundary condition of emotional resources is less investigated. Indeed, emotional resources are almost seen as an important antecedent variable on positiv behaviors (Agnoli et al., 2019; Kun et al., 2019; Peláez-Fernández et al., 2021) and negative employee behaviors (Golonka et al., 2017; Fiorilli et al., 2019; Valente and Lourenço, 2020) in previous studies. Therefore, this research proposes that emotional resources will moderate the relationship between emotional engagement and emotional exhaustion because a subordinate who has a higher level of emotional resources can optimize emotional resources to mitigate more emotional exhaustion by the effects of more emotional engagement.

## Literature Reviewing

**Figure 1:**
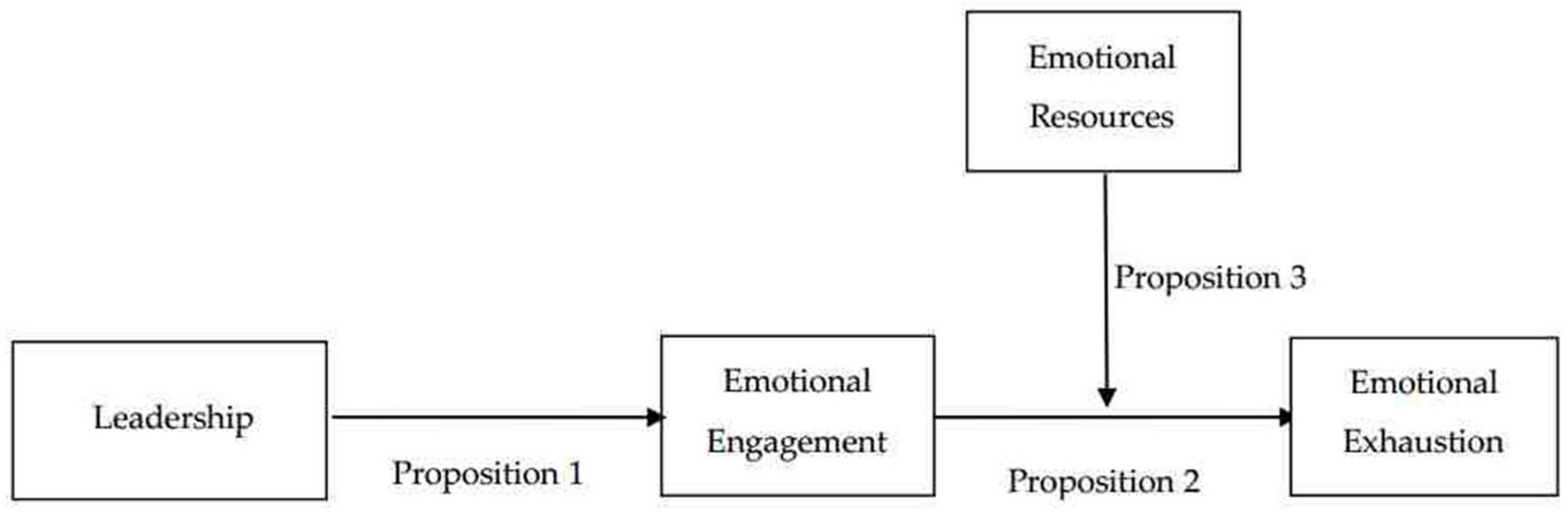
Emotional regulation model.

This research addresses an emotional regulation model to predict emotional exhaustion in Figure 1.

### Work Engagement Theory

In Kahn's engagement research, wok engagement represents “the simultaneous employment and expression of a person's preferred self in task behaviors that promote connections to work and to others, personal presence (physical, cognitive, and emotional) and active, full performances” (Kahn, 1990, p. 700). In short, employees will fall into work by investing personal energy into three dimensions of engagement (i.e., physical, cognitive, and emotional) (Kahn, 1990). For example, an engaged employee will put head (cognitive engagement), heart (emotional engagement), and body (physical engagement) into work to realize a high level of role performance. This research focuses on the emotional dimension of engagement because this research focuses on the domain of emotional regulation. Emotional engagement is defined by this research as a pleasure for work.

In addition, Kahn (1990) believes that whether an individual chooses to demonstrate work engagement depends on whether the individual feels the work environment is meaningful, safe, and confident or not. That is to say, meaningfulness, safety, and confidence are important antecedents of work engagement.

### Leadership and Emotional Engagement

An leader can modify a subordinate's value to meet a company value, and the company value can be delivered by an leader in the leadership process (Huang et al., 2021), which will align the subordinate's value with the company's value. In addition, a leader shows consideration and understanding for the subordinate, and it will let the subordinate feel that his or her work environment is safe and supportive. Finally, a leader shows respect for the subordinate, and it will make the subordinate feel confident. This research addresses proposition 1 as follows:

Hypothesis 1: Leadership can increase emotional engagement.

### Emotional Engagement and Emotional Exhaustion

Emotional exhaustion means a feeling of exhaustion for emotional resources (Maslach et al., 2001). Thus, an individual with a high level of emotional exhaustion suffers from feelings of fatigue, and he or she believes that his or her performance has suffered accordingly (Alsalhe et al., 2021). In contrast, emotional engagement is when an individual harnesses his or her full self into role performance by driving personal energy into emotional engagement, and the individual is connected, integrated, and focused on role performances. Therefore, an engaged employee must have abundant emotional resources to achieve role performance, so it must be able to reduce the situation of emotional exhaustion. This research addresses proposition 2 as follows:

Hypothesis 2: Emotional engagement can decrease emotional exhaustion.

### The Moderating Role of Emotional Resources

Emotional resources mean occupation-specific emotional resources (de Jonge et al., 2004; Yen, 2022). An individual with a high level of emotional exhaustion denotes that the individual has insufficient resources to deal with the job demand. Emotional resources can moderate the relationship between emotional engagement and emotional exhaustion because emotional resources are occupation-specific emotional resources that can boost the effects of emotional engagement on the status of emotional resource exhaustion. Based on conservation of resources theory (Hobfoll and Shirom, 2001), an individual employs personal resources to handle negative situations and the individual further seeks new resources to satisfy new job demands. Once personal resources are exhausted, emotional resources are additional resources, thereby supporting the moderating role of emotional resources. Indeed, lower emotional resources will yield higher-level stress for an employee, because the employee has a lower emotional resource. This research addresses proposition 3 as follows:

Hypothesis 3: Emotional resources can moderate the relationship between emotional engagement and emotional exhaustion.

## Discussion

This research adopts work engagement theory (Kahn, 1990) to address an emotional regulation model that describes how leadership affects emotional engagement and emotional exhaustion. The past study doesn't explore the mechanism of leadership on these variables, and thus this research provides incremental contributions to these fields.

The previous study also ignores how emotional resources can moderate the relationship between emotional engagement and emotional exhaustion, so this research also provides an incremental contribution to the literature on emotional resources. Indeed, emotional resources should be seen as a moderator because they cannot only increase positive employee behaviors but also decrease negative employee behaviors.

Finally, contemporary firms must deal with negative employee behaviors to realize competitive advantages, and this research addresses leadership as an optimal path. Indeed, leadership is a good thing to guide employees toward positive attitudes, so these employees must show high performance and satisfaction. This research thus suggests that firms should incorporate leadership into education training courses to improve supervisors' leadership ability.

## Author Contributions

All authors listed have made a substantial, direct, and intellectual contribution to the work and approved it for publication.

## Conflict of Interest

The authors declare that the research was conducted in the absence of any commercial or financial relationships that could be construed as a potential conflict of interest.

## Publisher's Note

All claims expressed in this article are solely those of the authors and do not necessarily represent those of their affiliated organizations, or those of the publisher, the editors and the reviewers. Any product that may be evaluated in this article, or claim that may be made by its manufacturer, is not guaranteed or endorsed by the publisher.
